# Rapid Eukaryotic Impedimetric Biosensing of Naproxen and Isoniazid: A Proof-of-Concept for Acute Toxicity Monitoring

**DOI:** 10.3390/bios16050298

**Published:** 2026-05-20

**Authors:** Zala Štukovnik, Nik Perko, Urban Bren

**Affiliations:** 1 Faculty of Chemistry and Chemical Engineering, University of Maribor, Smetanova Ulica 17, 2000 Maribor, Slovenia; zala.stukovnik1@um.si (Z.Š.); nik.perko1@student.um.si (N.P.); 2 Faculty of Mathematics, Natural Sciences and Information Technologies, University of Primorska, Glagoljaška Ulica 8, 6000 Koper, Slovenia; 3 Institute of Environmental Protection and Sensors, Beloruska Ulica 7, 2000 Maribor, Slovenia

**Keywords:** acute toxicity monitoring, impedimetric biosensor, *Saccharomyces cerevisiae*, electrochemical impedance spectroscopy (EIS), pharmaceutically active compounds, naproxen, isoniazid

## Abstract

This study presents a rapid, eukaryotic impedimetric biosensor that applies the yeast *Saccharomyces cerevisiae* as a robust, cost-effective biorecognition element for monitoring the acute toxicity of two representative pharmaceuticals, naproxen and isoniazid, in aquatic systems. The biosensor utilizes a previously developed three-electrode system made from type 316 stainless steel. Yeast cells seeded onto these electrodes serve as the biosensing element. By monitoring changes in electrical impedance, the system quantifies the cellular stress induced by pharmaceutical exposure. Electrochemical Impedance Spectroscopy (EIS) revealed a concentration-dependent decrease in both resistance and capacitance, attributed to cell death and subsequent desorption from the working electrode surface. These findings were validated through optical density at 600 nm (OD_600_) growth curve analysis and methylene blue viability staining, which confirmed metabolic inhibition and membrane damage. Results indicate a linear response for naproxen within the 2.5 mM to 20 mM range, with a LOD of 0.509 mM, and for isoniazid within the 10 mM to 100 mM range, with a LOD of 0.684 mM. Naproxen demonstrated a more pronounced cytotoxic effect, with cell viability dropping to 41.08% at 10 mM compared to 68.79% for isoniazid. While conventional analytical methods focus on chemical quantification, this proof-of-concept biosensor provides a rapid toxic/non-toxic signal, offering a biologically relevant tool for real-time monitoring of industrial waste streams and acute environmental contamination.

## 1. Introduction

Monitoring pharmaceutical residues in water and industrial wastewater is critical for environmental safety. This study presents a rapid eukaryotic impedimetric biosensor designed as a proof-of-concept for acute toxicity monitoring. Although typically present at trace concentrations ranging from ng/L to μg/L, these pharmaceutical contaminants persist in surface water, groundwater, and drinking water, posing substantial health risks and potentially bioaccumulating in nontarget organisms [1,2]. The need to monitor related biological effects has driven demand for biosensors, analytical devices that are not only cost-effective and time-efficient but also capable of measuring the specific impact of chemical stressors, a capability often lacking in conventional analytical methods such as HPLC-MS [3,4,5]. We use the yeast *Saccharomyces cerevisiae* as a robust, cost-effective biorecognition element to detect the physiological effects of two common pharmaceuticals, naproxen and isoniazid. The selection of naproxen and isoniazid was strategically based on their representation of two distinct chemical and pharmacological classes: a nonsteroidal anti-inflammatory drug (NSAID) and a primary antibiotic, respectively [6,7]. These compounds were chosen not only for their high global consumption and partial metabolism, resulting in significant environmental discharge, but also for their different toxicity mechanisms. Naproxen is widely recognized for its persistence and tendency to form even more toxic oxidative transformation products in the environment [8]. Isoniazid, meanwhile, poses a unique risk by potentially promoting the development of antibiotic-resistant strains of *Mycobacterium tuberculosis* in aquatic systems [9]. Together, they serve as ideal model analytes to demonstrate the biosensor’s versatility in detecting diverse physiological stressors in eukaryotic systems. Naproxen ((S)-(+)-2-(6-methoxy-2-naphthyl) propionic acid) represents a nonsteroidal anti-inflammatory drug (NSAID) used to relieve pain and inflammation in conditions such as arthritis, tendinitis, and gout [10,11,12]. It is one of the most widely used drugs in the world [9], as it is available in pharmacies without a prescription [13], and its widespread application in recent years has contributed to its increasing presence in the environment, where it poses a risk to the health of aquatic and terrestrial organisms [7]. It enters the environment through domestic and hospital wastewater and, in some cases, even through wastewater treatment plant effluents [11], where studies have shown that conventional wastewater treatment methods, including phototransformation and advanced oxidation processes [14], remain ineffective in its removal, as they can lead to greater toxicity in the environment via the formation of more toxic oxidative products [15,16]. Compared to other pharmaceutical active substances, biodegradation in the environment is also more difficult [17]. Naproxen has been detected in all types of water, including drinking water and groundwater. Despite low concentrations in the ng/L to μg/L range, long-term exposure of nontarget organisms to naproxen, particularly when combined with other active substances, can lead to various adverse effects [8,18]. Studies have shown that naproxen exhibits cardiovascular toxicity, hepatotoxicity, nephrotoxicity, mutagenicity, and causes oxidative stress and gastrointestinal damage [17,19].

Isoniazid (pyridine-4-carbohydrazide) is an antibiotic used as a first-line medication in the prevention and treatment of tuberculosis, a disease caused by *Mycobacterium tuberculosis*, with which roughly a third of the world’s population has been infected [20,21]. Despite its beneficial effects, isoniazid is only partially metabolized after oral administration, and about 75% is excreted in its unmodified form into water systems [22,23,24]. This can lead to contamination of the aquatic environment and the development of antibiotic-resistant strains of *Mycobacterium tuberculosis*, as reported by the World Health Organization on the growing number of new infections and reinfections of tuberculosis [25]. To remove isoniazid from wastewater, it must be treated in sewage treatment plants, which are often unavailable in developing countries. An additional problem is the widespread practice of discharging wastewater directly into water bodies or onto cultivated land, which poses significant health, economic, and environmental risks [26,27]. Although its toxicity and presence in the aquatic environment have been poorly studied, known adverse effects include neuropathy, hepatotoxicity, rhabdomyolysis, and acidosis [28,29].

To address the limitations of conventional detection methods, which are expensive, time-consuming, and unable to measure the biological effects of chemical stressors, this study utilizes an impedimetric biosensor [30,31]. While previous investigations have successfully applied cell-based sensors for contaminants, such as HepG2 cells for isoniazid detection, these often require complex handling [32]. In contrast, *Saccharomyces cerevisiae* yeast cells were selected as the biorecognition element for this study due to their robustness, cost-effectiveness, and ability to respond to physiological stimuli without the need for sophisticated sterile techniques [33]. Moreover, *Saccharomyces cerevisiae* serves as a model system for eukaryotic cell biology, and in our biosensor, it can serve as a biorecognition element that indicates whether a compound is toxic or non-toxic [34]. While conventional methods can identify the presence and concentration of a pharmaceutical, they do not provide immediate data on whether the wastewater is actually toxic to a eukaryotic system (Table 1).

The main principle of our impedimetric biosensor is to directly correlate the biological effect of the pharmaceutical with an impedance signal. Electrochemical impedance spectroscopy (EIS) monitors the adhesion layer of yeast cells on the working electrode surface [39]. Upon exposure to varying concentrations of naproxen or isoniazid, the cells undergo dose-dependent stress, leading to desorption and death [40]. This biological response results in a measurable decrease in the system’s electrical resistance and overall impedance [40,41,42]. To validate these effects, optical density (OD_600_) measurements were used to generate growth curves, enabling quantification of changes in cell growth inhibition and death [43].

Exposing yeast cells to varying concentrations of naproxen was expected to induce a dose-dependent physiological stress, leading to cell desorption, which can be quantified as a concentration-dependent decrease in impedance by the impedimetric biosensor. By directly linking cell viability to impedance changes, this approach can provide a rapid, reliable, and biologically relevant method for monitoring the biological effects of pharmaceutical contaminants in aquatic environments.

The aim of this study was to use a previously optimized biosensor to detect naproxen and isoniazid, representative pharmaceutical compounds, by quantifying their specific inhibitory effects on *Saccharomyces cerevisiae*.

## 2. Materials and Methods

### 2.1. Materials

Both the design and configuration of the electrochemical cell were previously tested and optimized by Štukovnik et al. [42]. The biosensor consisted of an electrochemical cell with three identical steel electrodes in a previously optimized RCW side-by-side configuration. The three-electrode electrochemical cell consisted of a working electrode (WE) with *Saccharomyces cerevisiae* cells as the biorecognition element on its surface, a counter electrode (CE), and a reference electrode (RE). The biosensor is designed to measure the relative difference between the electrode covered with a biological layer (WE) and the one that is not (RE), while CE provides the current flow. The electrodes measuring 20 mm × 5 mm were made from 0.050 mm thick type 316 stainless steel (M-Tech^®^F, manufacturer Georg Martin GmbH, Dietzenbach, Germany). 8.72 g (wet weight) of yeast cells, *Saccharomyces cerevisiae* (commercial fresh yeast (Fala fresh yeast, Lesaffre, Ljubljana, Slovenia)), were mixed with 2 mL of 0.9% NaCl to prepare a viscous mixture. A biorecognition element was applied to a 5 mm × 5 mm area on the WE. The fixation side of the electrodes was insulated using vinyl tape, and the system was sealed with glass. Chemicals isoniazid (C_6_H_7_N_3_O, Sigma-Aldrich, (St. Louis, MO, USA) ≥99%, CAS: 54-85-3, M = 137.14 g/mol) and naproxen (CH_3_OC_10_H_6_CH(CH_3_)CO_2_H, Sigma-Aldrich, CAS: 22204-53-1, M = 230.26 g/mol) were used to prepare solutions of varying concentrations and served as analytes. A 0.9% NaCl solution (saline) was prepared using Sigma-Aldrich NaCl (≥99.0%, CAS:7647-14-5, M = 58.44 g/mol) and MilliQ water (Merck KGaa, Darmstadt, Germany). A positive control, 10 mM H_2_O_2_ (CAS: 7722-84-1, 34.01g/mol was prepared in 0.9% NaCl. A saline solution was then applied to measure blanks and prepare analyte solutions. The 2.5 mM, 5 mM, 7.5 mM, 10 mM, and 20 mM solutions of naproxen (Sigma-Aldrich, CAS: 22204-53-1, M = 230.26 g/mol) and 10 mM, 25 mM, 50 mM, 75 mM, and 100 mM solutions of isoniazid (Sigma-Aldrich, ≥99%, CAS: 54-85-3, M = 137.14 g/mol) were prepared for the measurements. A schematic representation of the biosensor is shown in the Supplementary Materials as Figure S1.

The MultiPalmSens4 potentiostat, used to perform EIS measurements of naproxen and isoniazid solutions, was controlled by the program PSTrace for data collection and interpretation.

For measuring OD_600_, a sterile YPD culture medium (Yeast Extract Peptone Dextrose) was prepared, consisting of 5 g Meat peptone (Sigma-Aldrich, CAS: 91079-38-8), 3 g Yeast extract (Sigma-Aldrich, CAS: 8013-01-2), 3 g Malt extract (Sigma-Aldrich, CAS: 8002-48-0), 10 g D(+) glucose (Sigma-Aldrich, CAS: 50-99-7) and 1000 mL MilliQ water, which was then processed in an autoclave for 20 min at 121 °C and 1 bar. The Synergy H1 microplate reader (BioTek Instruments, Inc., Winooski, VT, USA) was utilized to monitor OD_600_ changes over time, along with BioTek Gen5 for data collection and interpretation.

### 2.2. Methods

**Figure 1:**
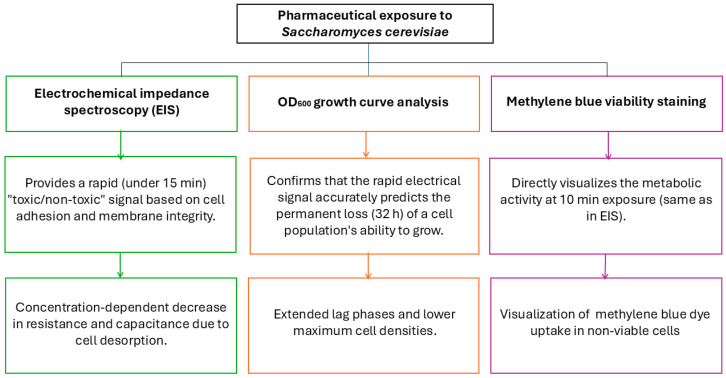
Workflow diagram for measurements of pharmaceutical exposure to *Saccharomyces cerevisiae*.

When evaluating the correlation between short-term impedimetric signals and long-term growth inhibition, it is essential to account for the nature of cellular stress. The 10 min EIS exposure in saline serves as a rapid probe of immediate membrane integrity and cell adhesion to the electrode. These initial biophysical changes are early indicators of toxicity. By contrast, the 32 h growth curve analysis in YPD demonstrates that the physiological stress detected during the initial minutes of exposure has a lasting impact on the yeast’s ability to recover and proliferate (Table 1). The extended lag phases and lower maximum cell densities observed in the growth curves confirm that the immediate impedance drop is not a transient fluctuation but a reliable predictor of long-term biological inhibition. Thus, the two methods provide complementary data; EIS offers a rapid diagnostic signal of acute toxicity, while OD_600_ measurements provide biological validation of the severity and persistence of that toxic effect. While methylene blue viability staining directly visualized activity at 10 min of exposure, the same time as in EIS measurements (Figure 1).

#### 2.2.1. Electrochemical Measurements

**Figure 2:**
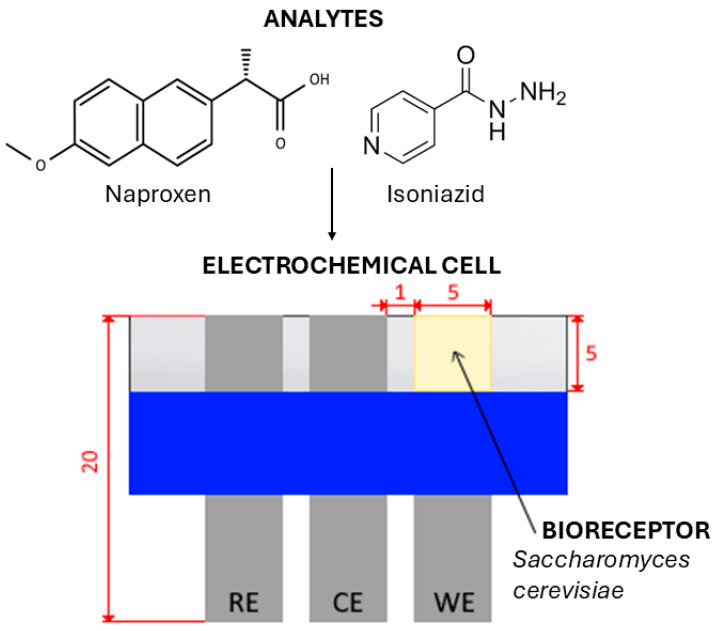
The biosensor, with an electrochemical cell in an RCW side-by-side configuration, where the analytes are Naproxen and Isoniazid, and the bioreceptor is a layer of *Saccharomyces cerevisiae* cells.

**Figure 3:**
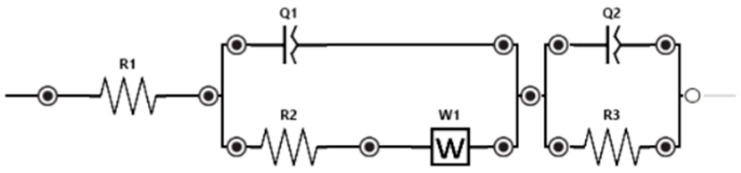
Equivalent electrical circuit (EEC).

The procedure for measuring was analogous to that described by Štukovnik et al. [33]. The electrochemical cell, with *Saccharomyces cerevisiae* as the biorecognition component (Figure 2), was connected to a MultiPalmSens4 potentiostat. Measurements were conducted at the open-circuit potential (OCP), which was monitored for 10 min prior to each session to ensure system equilibrium. The stabilized potential obtained during this time period was subsequently applied as the bias condition for all further Electrochemical Impedance Spectroscopy (EIS) measurements. To maintain experimental consistency, the same sensor was used sequentially for measurements across all analyte concentrations. Each EIS measurement lasted 180 s; this relatively short duration ensured that the biorecognition layer’s stability was not compromised during sensing. Carryover between concentrations was prevented by sequentially injecting increasing concentrations into the established electrochemical cell. The Equivalent Electric Circuit (EEC), depicted in the full electrochemical setup schematic (Figure 3) was applied to model the electrolyte resistance, the yeast adhesion layer, and the electrode surface processes. The fitting procedure and validation were performed transparently using the PSTrace 5.8 software environment, which employed its built-in fitting algorithms to correlate the impedimetric response with the physiological state of *Saccharomyces cerevisiae* cells. 1 mL of 0.9% NaCl was injected into the system, and the EIS of the blank was measured after 10 min. Following this, 1 mL of naproxen or isoniazid at a specific concentration was injected into the system, and EIS measurements were repeated for each concentration, with a 10 min waiting period after the addition of the pharmacological compound. The EIS technique was applied to obtain information regarding the adhesive layer of *Saccharomyces cerevisiae* yeast cells on the stainless steel surface of the WE, by tracking the system’s response to a low amplitude sinusoidal signal in a frequency range from 10 kHz to 100 mHz with a current range from 100 pA to 10 mA, 10 points per decade, and a potential amplitude of 40 mV. Each EIS measurement was repeated 3 times, and Nyquist and Bode diagrams were interpreted for each naproxen and isoniazid concentration. The 180-s EIS measurement, following a brief 10 min stabilization (OCP measurement), allows for a total “sample-to-signal” time of less than 15 min, making it suitable for early-warning systems in industrial runoff.

#### 2.2.2. Growth Curve Analysis

Growth curve analysis was performed through OD_600_ measurements. The yeast suspension was diluted to an initial OD_600_ of 0.1 before being added to the microplate. For the negative control, 150 μL of *Saccharomyces cerevisiae* cells in YPD medium, along with 50 μL of 0.9% NaCl, was measured. Changes in optical density over time were monitored for three low, medium, and high concentrations of naproxen (2.5, 5, and 10 mM), as well as isoniazid (10, 25, and 50 mM). The highest concentrations used in EIS (20 mM naproxen and 100 mM isoniazid) resulted in such severe inhibition that they were not applied in growth curve modeling, since they became less informative. These solutions were mixed with 150 μL of *Saccharomyces cerevisiae* cells in YPD medium and 50 μL of analytes at four times the desired concentrations to achieve the specified final concentrations. The OD_600_ of each well was measured over 32 h at 25 °C, with 30 min intervals, using constant orbital mixing at 200 rpm to ensure homogenization and prevent sedimentation.

#### 2.2.3. Methylene Blue Viability Staining

Cell viability determination is the most commonly applied method for assessing the effects of various stressors in toxicity research [27]. A yeast cell suspension was prepared using compressed fresh yeast Fala by dissolving 1 g of the biomass in 10 mL of 0.9% NaCl to form an initial stock. Because the initial stock was highly concentrated, it was homogenized by vortexing at 3000 rpm and subsequently diluted 1:10 to achieve a cell density suitable for counting. Viability was assessed using the methylene blue exclusion method, for which a 0.1% *w*/*v* methylene blue solution was prepared by dissolving 0.1 g of methylene blue in 100 mL of distilled water. Cells were treated with various concentrations of naproxen and isoniazid for 10 min before analysis. Equal volumes of the diluted yeast suspension and the methylene blue solution were mixed (1:1) and incubated for 3 to 5 min. The incubation time was strictly monitored to avoid false positives due to exposure exceeding 10 min. The stained samples were examined under an optical microscope at 400× magnification. Colorless cells were classified as viable, and blue cells were classified as non-viable.

#### 2.2.4. Statistical Analysis

Following EIS measurements, which were performed three times (*n* = 3) for each concentration of naproxen and isoniazid (with 0.9% NaCl as the blank solution), the mean value (MV), standard deviation (SD), and accuracy of each measurement were calculated. Calibration curves were fitted for both naproxen and isoniazid, and the 3.3-Sigma criteria were used to determine the LOD for each compound. The accuracy values presented in Table 2 and Table 3 represent the signal precision for each analyte. This was calculated by expressing the standard deviation as a percentage of the mean (coefficient of variation) and subtracting that percentage from 100%. The calibration curves for both naproxen and isoniazid were constructed by plotting the logarithm of the mean absolute impedance (|Z|) against the logarithm of the analyte concentration. The specific response variable was obtained from the system’s Bode plots at a low frequency of 10^−0.9^ Hz. This frequency was selected because it allows for a clear differentiation of impedance changes associated with the yeast adhesion layer and subsequent desorption from the electrode surface. For each analyte concentration, three separate EIS measurements were performed to calculate the mean absolute impedance and the corresponding standard deviation, ensuring the reliability and accuracy of the resulting linear models.

## 3. Results

### 3.1. EIS Measurements

For each pharmaceutical compound, naproxen and isoniazid, the results of electrochemical impedance spectroscopy were presented as Nyquist and Bode diagrams. In the Nyquist diagram, the results were shown as the real (resistance) and imaginary (capacitance) components of the system’s impedance response upon addition of the active substance, providing information on mass transport processes (lower frequencies) and charge transfer (higher frequencies). In the Bode diagram, the results were presented simultaneously as two spectra: impedance and phase, showing the dependence of the system’s absolute impedance (∣Z∣) and phase angle on frequency. Their change relative to the concentration of the pharmaceutical compound thus provides information about the system’s resistance, capacitance, and diffusion, as well as its responsiveness to the active substance. An equivalent electric circuit, determined for each pharmaceutical compound (Figure 3), described the processes within the closed electrochemical system.

In the circuit, R1 (resistance) represents the electrolyte resistance; Q1 (capacitance) and R2 represent the yeast layer; and Q2, W1 (Warburg impedance), and R3 represent the processes on the working electrode. 

A biosensor based on *Saccharomyces cerevisiae* was tested with 10 mM H_2_O_2_, serving as a positive control toxicant to verify the system’s sensitivity to acute cellular stress.

**Figure 4:**
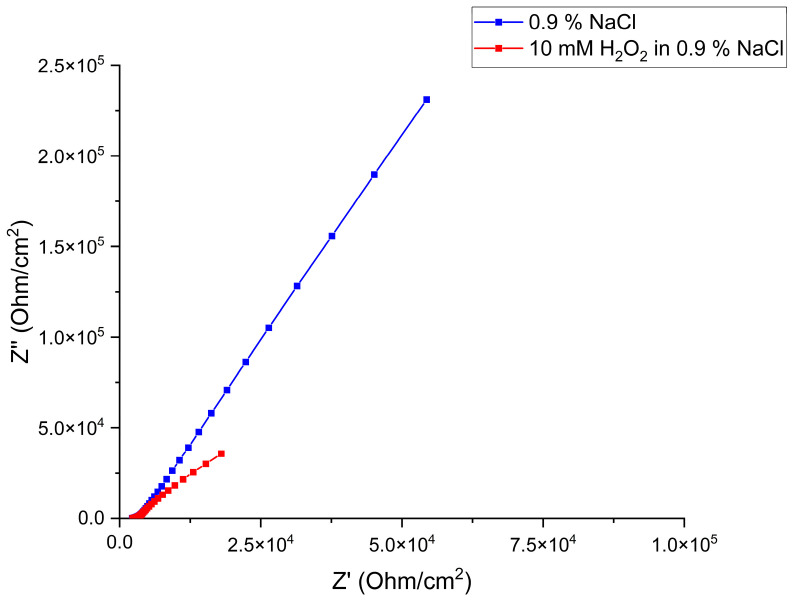
Nyquist diagram of the impedimetric biosensor response to 10 mM H_2_O_2_ in 0.9% NaCl (red) compared to a 0.9% NaCl (blue).

As shown in the Nyquist plot (Figure 4), exposure to 10 mM H_2_O_2_ results in a significant decrease in both the real (resistance) and imaginary (capacitance) components of impedance. This decrease in impedance suggests that the insulating capacity of the *Saccharomyces cerevisiae* layer is compromised.

**Figure 5:**
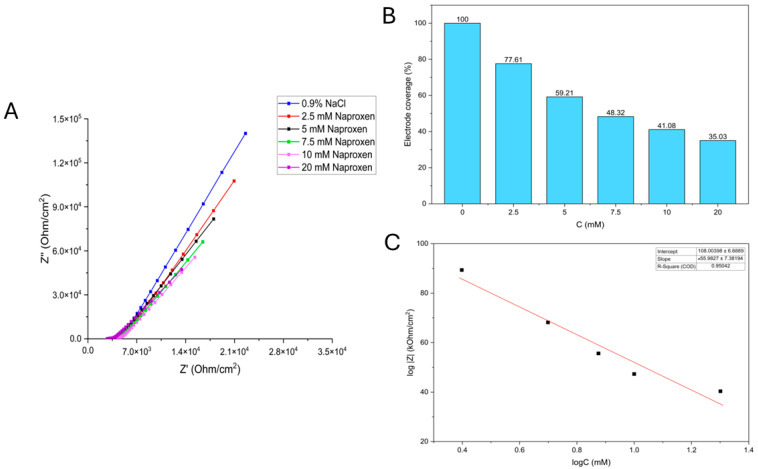
(**A**) Nyquist diagram showing the system’s response to the addition of 0.9% NaCl (blue curve) and naproxen solutions of varying concentrations (depicted by differently colored curves); (**B**) electrode coverage (%); (**C**) the calibration curve.

**Figure 6:**
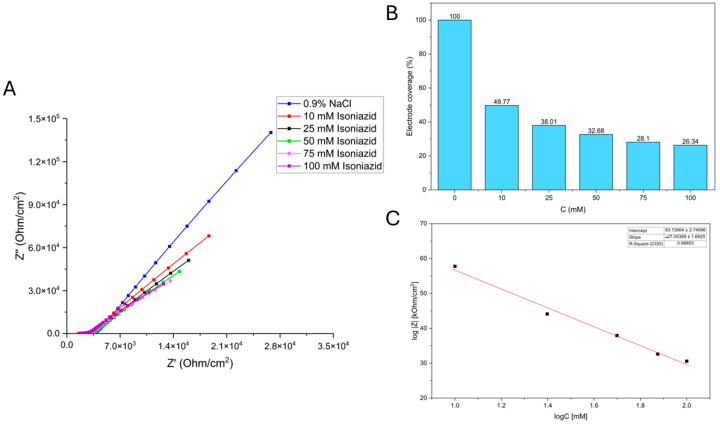
(**A**) Nyquist diagram showing the system’s response to the addition of 0.9% NaCl (blue curve) and isoniazid solutions of varying concentrations (represented by differently colored curves); (**B**) electrode coverage (%); (**C**) the calibration curve.

Electrode coverage (%) presented in Figure 5B and Figure 6B represents the relative biological insulation of the working electrode (WE) provided by the *Saccharomyces cerevisiae* adhesion layer. It was calculated using the absolute impedance (Z) values obtained from the Bode plots at a specific frequency. The blank measurement, conducted in a 0.9% NaCl solution before the addition of any pharmaceutical analyte, served as the reference for 100% coverage. At this stage, the yeast layer is at its maximum density and provides the highest level of biological insulation. To determine coverage for each analyte concentration, the absolute impedance value (|Z|) for each Bode plot was divided by the absolute impedance of the control (blank) and multiplied by 100% to yield a percentage. A decrease in this percentage directly quantifies physiological stress and subsequent cell death or desorption from the electrode surface, as the loss of cells allows the conductive electrolyte to more easily access the stainless-steel surface, thereby reducing the system’s overall impedance.

EIS measurements were performed in triplicate (*n* = 3), ensuring that the mean values and standard deviations reported for both naproxen and isoniazid reflect a consistent, repeatable electronic response. The precision of these signals is evidenced by accuracy values consistently exceeding 90% across all tested concentrations, with linear correlation coefficients (R^2^) reaching above 0.950 (naproxen) to 0.988 (isoniazid).

#### 3.1.1. Naproxen

The reduction in the system’s impedance with increasing naproxen concentrations is illustrated in a bar chart (Figure 5B). The rate of decrease appears to slow down as the concentration increases. At the highest concentration shown (20 mM), the impedance ratio has reduced to 35.03% of its original value.

The calibration curve for naproxen, depicted in Figure 5C, illustrates the relationship between the logarithm of the system’s absolute impedance and the logarithm of the naproxen solution concentration. The system’s linearity was established over a concentration range of 2.5 mM to 20 mM, yielding an R^2^ of 0.950.

In Table 1, the mean absolute impedance of the system (Mean log|Z|) decreases with increasing naproxen concentration, and the standard deviation (SD), as well as accuracy (above 90%), are reported.

#### 3.1.2. Isoniazid

The Nyquist diagram is shown in Figure 6A, which illustrates the system’s response after the addition of 0.9% NaCl (represented by the blue curve) and isoniazid solutions of varying concentrations (colored differently). The decrease in system impedance with increasing isoniazid concentration is shown in a bar chart (Figure 6B), confirming isoniazid’s influence on system impedance. Using the calibration curve (Figure 6C), the relationship between the logarithm of the system’s absolute impedance and the logarithm of the isoniazid solution concentration is displayed. Our findings confirmed that impedance decreases with increasing isoniazid concentration. The linearity of the system was established within a concentration range of 10 mM to 100 mM, yielding an R^2^ value of 0.988. Using the 3.3 − Σ criterion, the LOD was calculated to be 0.684 mM. Similar to the findings with naproxen, the calibration curve shows a successful linear fit despite greater data variability, allowing us to conclude that the prepared biosensor can indeed detect isoniazid in solution.

In Table 2, the decrease in the system’s mean absolute impedance with increasing isoniazid solution concentration is presented. The standard deviation (SD) and accuracy (above 90%) are reported.

### 3.2. Growth Curve Analysis

**Figure 7:**
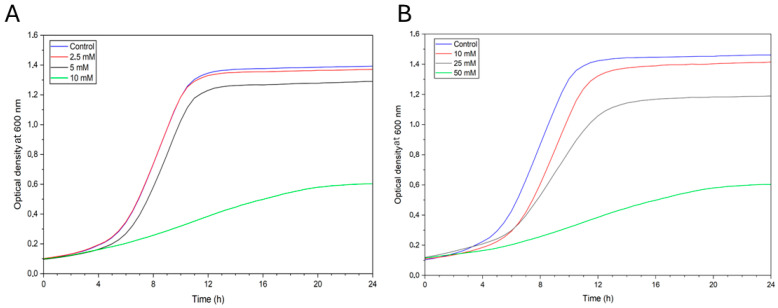
Effect of naproxen (**A**) and isoniazid (**B**) on the growth curves of *Saccharomyces cerevisiae* measured by optical density at 600 nm. Figure 7 presents the growth curves of yeast cells of the species *Saccharomyces cerevisiae*, specifically their responses to the addition of 0.9% NaCl (blue curve) and to increasing concentrations of naproxen (**A**) and isoniazid (**B**) as differently colored curves.

Growth curve analysis (OD_600_) was used to measure inhibition caused by various concentrations of naproxen and isoniazid (Figure 7).

### 3.3. Optical Microscopy

Cell viability was determined using the methylene blue exclusion assay, with results derived from three independent biological replicates (*n* = 3). For each concentration, control cells were analyzed across multiple random fields of view and blindly counted to ensure representative sampling. Data are expressed as the mean percentage of viable cells relative to the total population, with statistical variability represented by the standard deviation (SD). To confirm the significance of the observed dose-dependent toxicity, a t-test was performed, comparing each treatment group to the untreated control.

**Figure 8:**
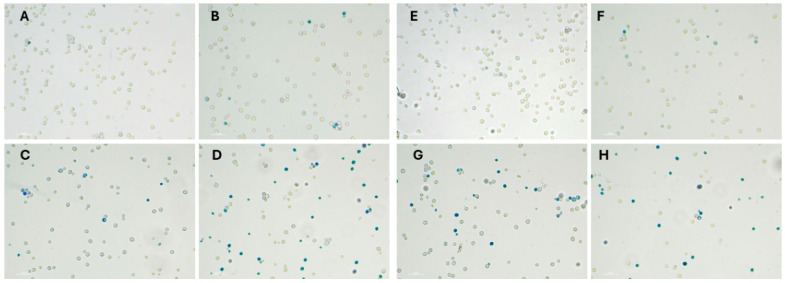
A concentration-dependent cytotoxic effect of naproxen: (**A**) negative control; (**B**) 2.5 mM naproxen; (**C**) 5 mM naproxen; (**D**) 10 mM naproxen and isoniazid; (**E**) negative control; (**F**) 10 mM isoniazid; (**G**) 25 mM isoniazid; (**H**) 50 mM isoniazid. Blue-stained cells represent non-viable cells.

Figure 8A,E represent negative controls and show a high cell density with very few stained (dead) cells. Figure 8B represents 2.5 mM naproxen, where the cell density appears slightly lower than in A, but the majority of cells remain viable. Figure 8C represents 5 mM naproxen, where a noticeable increase in the number of blue-stained cells occurs. Small clusters of dead cells are forming, indicating moderate toxicity. Figure 8D represents 10 mM naproxen, with a significant increase in the proportion of blue-stained cells. A large percentage of the population has taken up the dye, indicating extensive cell death at this highest concentration.

Figure 8E–H demonstrates a concentration-dependent cytotoxic effect of isoniazid on yeast cells. As the concentration increases from 10 mM (Figure 8E) to 50 mM (Figure 8H), a clear progression from high viability to high mortality is observed.

**Figure 9:**
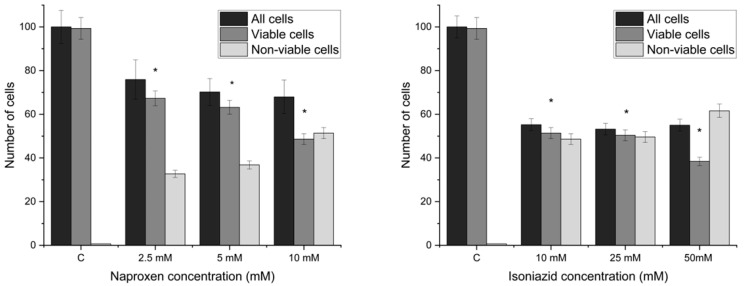
Representation of results from optical microscopy (asterisk presents a significant difference with respect to the untreated control cells (* equals *p* < 0.05; *t*-test). The black box represents both living and dead cells, the gray living cells, and the white dead cells.

The results from the optical microscopy analysis demonstrate a significant, dose-dependent reduction in cell viability following treatment. As shown in the data, all concentrations of both naproxen and isoniazid differ significantly from the untreated control (*p* < 0.05; *t*-test). The most pronounced effect was observed at the 50 mM isoniazid concentration, which produced the lowest percentage of viable cells and a notable decline in the total cell population (black box). The trend observed in Figure 9 suggests that both naproxen and isoniazid induce cell death.

## 4. Discussion

Unlike conventional methods that measure concentration, this method detects the physiological stress the contaminant imposes on a biological system. There is a clear trend: increasing concentrations of both naproxen and isoniazid decrease the system’s resistance and capacitance. This is attributed to cell death and desorption from the *Saccharomyces cerevisiae* working electrode. The growth curves validate the EIS findings, where higher concentrations resulted in longer lag phases and lower maximum cell densities, confirming that the impedance drop correlates with cell inhibition or death. Naproxen appeared to have a more significant effect on the cells than isoniazid, with larger decreases in impedance components and a more pronounced growth inhibition. The variation in capacitance measurements between the control and treated samples reflects changes in the thickness of the yeast cell layer on the working electrode (WE). In the control solution (0.9% NaCl), the Bode diagram (Figure S2) initially identified electrolyte resistance at high frequencies, while at lower frequencies, a phase angle of 81° indicated a strongly capacitive system due to the thick, insulating layer of healthy yeast cells. However, as naproxen concentrations increased from 2.5 mM to 20 mM, both resistance and capacitance decreased. This shift was marked by a decrease in the phase angle from 81° to 74° at low frequencies and from 28° to 15° at medium frequencies. These changes, coupled with the overall decline in absolute impedance, suggest that naproxen-induced stress led to cell death and subsequent desorption from the electrode surface, thereby reducing the biological barrier. The calibration curve in Figure 5C and the box chart in Figure 5B clearly indicate that the system’s impedance decreases with increasing naproxen concentration. Using the 3-Sigma criterion, the limit of detection (LOD) was determined to be 0.509 mM. Consistent with the trends observed for naproxen, the addition of isoniazid at concentrations ranging from 10 mM to 100 mM resulted in a progressive decrease in both the resistance and capacitance of the electrochemical system. This impedimetric shift indicates that higher isoniazid concentrations induce significant yeast cell mortality and subsequent desorption, thereby reducing the biological insulation of the working electrode and granting the electrolyte greater access to the SS316 surface. The linearity of the system for isoniazid effect detection was established within a concentration range of 10 mM to 100 mM, yielding an R^2^ value of 0.988. Using the 3-Sigma criterion, the LOD was calculated to be 0.684 mM. These results confirm that the biosensor effectively translates pharmaceutical-induced stress into quantifiable changes in both optical density and electrical impedance.

The OD_600_ growth curves showed that 2.5 mM naproxen had a negligible impact on growth; 5 mM and 10 mM induced a concentration-dependent toxic response, characterized by an extended lag phase and a significantly lower OD_600_ value in the exponential phase. In the detection of the isoniazid effect, the curve following the addition of the 10 mM solution showed lower OD_600_ values than the control. A similar observation was made for the curves at 25 mM and 50 mM, which also exhibited less steep exponential phases and progressively lower OD_600_ values. As with naproxen, the maximum cell density (OD_600_) decreases, suggesting that increasing concentrations lead to a greater cell stress, reduced proliferation, and increased cell death. This allows us to conclude that the prepared biosensor can detect the effect of isoniazid and naproxen in a solution.

Overall, exposure to the analytes induces membrane damage, as confirmed by methylene blue staining and metabolic inhibition, as measured by OD_600_. Dead or stressed cells lose their adhesion and detach from the electrode surface, decreasing the system’s resistance as the electrolyte (0.9% NaCl) gains easier access to stainless steel working electrodes. The decrease in capacitance further supports the thinning of the biorecognition layer, as the more conductive electrolyte replaces the cell layer. The absolute impedance (Figure S3) at 10^−0.9^ Hz indicates that the electrode coverage drops to 18.7% following H_2_O_2_ exposure. The positive control demonstrates a much higher level of toxicity than equivalent concentrations of the two pharmaceuticals. The impedance decrease observed confirms that the biosensor identifies toxic events, providing a benchmark for interpreting the pharmaceutical results.

The study validates EIS results using optical density (OD_600_) growth curves, confirming that the signal change is due to biological inhibition (Table 4). Our study applies a *Saccharomyces cerevisiae*-based impedimetric biosensor for the first time to detect naproxen, a widely used NSAID that is difficult to remove with conventional wastewater treatment, and poses environmental risks, as well as isoniazid, an antibiotic that can promote the development of antibiotic-resistant bacteria. Moreover, it replaces the complex HepG2 cells used in previous isoniazid detection with *Saccharomyces cerevisiae* yeast cells, which represent an effective, robust, and low-cost biorecognition element that does not require sophisticated sterile techniques or complex media. While the biosensor can detect high-concentration events, such as industrial discharges of naproxen reaching 2132 µgL^−1^, it lacks the sensitivity required to monitor the much lower concentrations (<0.1 µg L^−1^) found in many surface waters [44]. While the sensor is not yet sensitive enough for trace monitoring in open surface waters, its value lies in rapid screening of industrial spillages or wastewaters, where immediate toxic/nontoxic signals are prioritized over low concentration quantification. Our biosensor represents a tool for the rapid detection of acute toxicity in industrial and sewage effluents. In these environments, pharmaceutical concentrations can easily reach mg/L levels during discharge events, making immediate biological assessment more critical than high-sensitivity chemical quantification. The 10 min response time offers a significant advantage over conventional 24 h bioassays, providing a ‘first-line’ alarm system for acute contamination. Moreover, the use of *Saccharomyces cerevisiae* ensures a robust, cost-effective platform that can operate in non-sterile industrial settings, translating complex cellular stress into a simple, quantifiable impedimetric signal. The novelty of this research lies in the integration of three distinct analytical perspectives, electrochemical (EIS), kinetic (OD_600_), and morphological (methylene blue staining), to quantify pharmaceutical toxicity using a robust eukaryotic model. While the current LOD is higher than typical environmental trace levels, we emphasize that this research serves as a proof-of-concept for acute toxicity screening, enabling rapid detection of high-concentration spills or industrial discharges where chemical levels can reach mg/L levels. Unlike HPLC, which measures chemical presence, our biosensor provides immediate data on whether the water is toxic to a eukaryotic model. A sensor capable of providing an immediate toxic/non-toxic signal is more valuable for emergency response than a delayed quantitative report. Ultimately, the robustness of the stainless-steel electrodes, combined with the resilience of *Saccharomyces cerevisiae*, suggests that this biosensor is a viable candidate for on-site, real-time monitoring of industrial waste streams, where rapid detection of acute toxicity is prioritized over quantification of trace contaminants.

## 5. Conclusions

Our study successfully demonstrates the use of a rapid, eukaryotic impedimetric biosensor for monitoring the acute toxicity of pharmaceuticals in aquatic systems. A pre-optimized electrochemical cell with *Saccharomyces cerevisiae* yeast cells serving as the biorecognition element was utilized to detect two emerging contaminants, naproxen and isoniazid. EIS revealed concentration-dependent decreases in resistance and capacitance for both pharmaceuticals, which were validated by OD_600_ and methylene blue staining, confirming that the impedance drop directly correlates with cell death, metabolic inhibition, and subsequent desorption from the electrode surface. Naproxen exhibited a more pronounced cytotoxic effect than isoniazid. Although current detection limits are higher than typical environmental trace levels, this proof-of-concept establishes the biosensor as a viable candidate for real-time, on-site monitoring of industrial waste streams, offering a rapid toxic/non-toxic signal that is essential for emergency response to acute contamination events. As a proof-of-concept, this study used a standardized 0.9% NaCl solution to characterize the biosensor’s fundamental response. Future research will focus on validating the system using spiked environmental samples, such as treated wastewater effluent and surface water, to evaluate the impact of matrix effects on signal sensitivity. Such studies will be crucial for transitioning this technology from a laboratory prototype to a functional tool for on-site industrial monitoring.

## Figures and Tables

**Table 1:** Comparison of our biosensor to other methods for naproxen and isoniazid detection.

Method	Target	Sensitivity (LOD)	Pros	Cons	Reference
SPE-HPLC (PDA)	Naproxen	0.1 µg/L	A simple method for wastewater monitoring can detect multiple NSAIDs simultaneously.	Requires large sample volumes (100 mL) and specific pH adjustments (pH 2.5).	[35]
Fluorescent sensor with MIP-Carbon Dots	Naproxen	0.03 µM	Highly selective and stable under UV degradation.	Requires precise encapsulation of carbon dots into silica pores.	[36]
SPE-LC-MS/MS	Naproxen	0.02–0.59 µg/mL	High sensitivity, excellent for trace amounts, and standards ensure high accuracy.	Requires expensive tandem mass spectrometry equipment and expert operation.	[37]
Fluorometric and paper-based colorimetric dual-mode sensor	Isoniazid	0.28 µM (Fluor)/4.0 µM(Color)	Offers both lab-grade fluorescence and a portable smartphone-based colorimetric option.	Relies on the chemical destruction of nanosheets, which may be sensitive to other reducing agents.	[38]
Impedimetric Biosensor	Naproxen and Isoniazid	0.509 mM (NPX)/0.684 mM (ISO)	Measures actual biological effect, cost-effective, and rapid “toxic/non-toxic” signal.	Limits of detection are in the millimolar (mM) range.	Current work

**Table 2:** Parameters for naproxen measurements.

C [mmol/L]	logC [mmol/L]	Mean log|Z| [kOhm/cm^2^]	SD [kOhm/cm^2^]	Accuracy [%]
0	/	115.139	7.337	93.628
2.5	0.40	89.365	6.546	92.675
5	0.70	68.169	5.814	91.471
7.5	0.88	55.632	3.758	93.245
10	1.00	47.303	3.021	93.614
20	1.30	40.337	2.304	94.288

**Table 3:** Parameters for isoniazid measurements.

C [mmol/L]	logC [mmol/L]	Mean log|Z| [kOhm/cm^2^]	SD [kOhm/cm^2^]	Accuracy [%]
0	/	116.045	4.253	96.335
10	1.00	78.104	5.892	91.940
25	1.40	44.105	4.371	90.090
50	1.70	37.925	3.573	90.579
75	1.88	32.613	2.608	92.003
100	2.00	30.561	1.464	95.210

**Table 4:** Comparison of linear range, LOD, OD_600_ values at 10 mM, and cell viability (%) at 10 mM of naproxen and isoniazid.

	Naproxen (NSAID)	Isoniazid (Antibiotic)
Linear Range	2.5–20 mM	10–100 mM
LOD	0.509 mM	0.684 mM
OD_600_ at 10 mM	0.5 (High inhibition)	1.3 (Low inhibition)
Cell viability at 10 mM	41.08%	68.79%

## Data Availability

Data is contained within the article or Supplementary Materials.

## Supplementary Materials

The following supporting information can be downloaded at: https://www.mdpi.com/article/10.3390/bios16050298/s1. Figure S1: Bode diagram for 10 mM H_2_O_2_; Figure S2: Bode diagram for naproxen; Figure S3: Bode diagram for isoniazid.

## Abbreviations

The following abbreviations are used in this manuscript:

CE	Counter Electrode
EEC	Equivalent Electric Circuit
EIS	Electrochemical Impedance Spectroscopy
HPLC-MS	High-Performance Liquid Chromatography–Mass Spectrometry
LOD	Limit of Detection
MV	Mean Value
NSAID	Nonsteroidal Anti-inflammatory Drug
OD_600_	Optical Density at 600 nm
RCW	Reference-Counter-Working three-electrode system
RE	Reference Electrode
SD	Standard Deviation
SS316	Type 316 Stainless Steel
WE	Working Electrode
YPD	Yeast Extract Peptone Dextrose (culture medium)

## References

[1] Patel M., Kumar R., Kishor K., Mlsna T., Pittman C.U., Mohan D. (2019). Pharmaceuticals of Emerging Concern in Aquatic Systems: Chemistry, Occurrence, Effects, and Removal Methods. Chem. Rev..

[2] Letsoalo M.R., Sithole T., Mufamadi S., Mazhandu Z., Sillanpaa M., Kaushik A., Mashifana T. (2023). Efficient detection and treatment of pharmaceutical contaminants to produce clean water for better health and environmental. J. Clean. Prod..

[3] Babu V.R.S., Patra S., Karanth N.G., Kumar M.A., Thakur M.S. (2007). Development of a biosensor for caffeine. Anal. Chim. Acta.

[4] Tothill I.E. (2001). Biosensors developments and potential applications in the agricultural diagnosis sector. Comput. Electron. Agric..

[5] Burcu Aydın E., Aydın M., Kemal Sezgintürk M. (2020). Biosensors and the evaluation of food contaminant biosensors in terms of their performance criteria. Int. J. Environ. Anal. Chem..

[6] Szarka A., Vnuková L., Keršňáková Z., Viktoryová N., Hrouzková S. (2024). Contamination with Pharmaceuticals in Aquatic Environment: Focus on Analytical Methodologies. Appl. Sci..

[7] Wojcieszyńska D., Guzik U. (2020). Naproxen in the environment: Its occurrence, toxicity to nontarget organisms and biodegradation. Appl. Microbiol. Biotechnol..

[8] Xu C., Niu L., Guo H., Sun X., Chen L., Tu W., Dai Q., Ye J., Liu W., Liu J. (2019). Long-term exposure to the non-steroidal anti-inflammatory drug (NSAID) naproxen causes thyroid disruption in zebrafish at environmentally relevant concentrations. Sci. Total Environ..

[9] Yimcharoen M., Saikaew S., Wattananandkul U., Phunpae P., Intorasoot S., Tayapiwatana C., Butr-Indr B. (2023). Mycobacterium tuberculosis Adaptation in Response to Isoniazid Treatment in a Multi-Stress System That Mimics the Host Environment. Antibiotics.

[10] Vuoriluoto M., Tammelin T., Orelma H. (2025). Understanding interactions of pharmaceutical pollutants with cellulosic materials. Cellulose.

[11] Ríos A.L.M., Gutierrez-Suarez K., Carmona Z., Ramos C.G., Oliveira L.F.S. (2022). Pharmaceuticals as emerging pollutants: Case naproxen an overview. Chemosphere.

[12] Tehrani E., Faraji A.R., Shojaei N., Shahinmehr S., Najafi A., Hekmatian Z., Tehrani Z., Bornas B. (2023). An overview of the characteristics, toxicity, and treatment methods for the degradation of pharmaceutically active compounds: Naproxen as a case study. J. Environ. Chem. Eng..

[13] Brutzkus J.C., Shahrokhi M., Varacallo M.A. (2025). Naproxen.

[14] Yang Y., Ok Y.S., Kim K.H., Kwon E.E., Tsang Y.F. (2017). Occurrences and removal of pharmaceuticals and personal care products (PPCPs) in drinking water and water/sewage treatment plants: A review. Sci. Total Environ..

[15] Worku A.K., Ayele D.W., Teshager M.A., Omar M., Yerkrang P.P., Elgaddafi R., Alemu M.A. (2025). Recent advances in wastewater treatment technologies: Innovations and new insights. Energy Rev..

[16] Hama Aziz K.H., Mustafa F.S., Karim M.A.H., Hama S. (2025). Pharmaceutical pollution in the aquatic environment: Advanced oxidation processes as efficient treatment approaches: A review. Mater. Adv..

[17] Bindu S., Mazumder S., Bandyopadhyay U. (2020). Non-steroidal anti-inflammatory drugs (NSAIDs) and organ damage: A current perspective. Biochem. Pharmacol..

[18] Rastogi A., Tiwari M.K., Ghangrekar M.M. (2021). A review on environmental occurrence, toxicity and microbial degradation of Non-Steroidal Anti-Inflammatory Drugs (NSAIDs). J. Environ. Manag..

[19] Qureshi I.Z., Razzaq A., Naz S.S. (2024). Testing of acute and sub-acute toxicity profile of novel naproxen sodium nanoformulation in male and female mice. Regul. Toxicol. Pharmacol..

[20] Sasu S., Metzger J., Kranert M., Kümmerer K. (2015). Biodegradation of the Antituberculosis Drug Isoniazid in the Aquatic Environment. Clean–Soil Air Water.

[21] Aquino de Queiroz J.L., Medeiros L.G., Augusto da Silva K., Fontes Galvão F.M., Oliveira do Nascimento J.H., Martínez-Huitle C.A., Castro P.S. (2023). Development of recycled and miniaturized electroanalytical sensor: Probing isoniazid determination in environmental water matrices. Chemosphere.

[22] Wang P., Pradhan K., Zhong X.B., Ma X. (2016). Isoniazid metabolism and hepatotoxicity. Acta Pharm. Sin. B.

[23] Sankar J., Chauhan A., Singh R., Mahajan D. (2024). Isoniazid-historical development, metabolism associated toxicity and a perspective on its pharmacological improvement. Front. Pharmacol..

[24] Khalid N.K., Reyaroth M.P., Devadasan D., Aravind U.K., Aravindakumar C.T. (2022). Sonochemical Degradation Studies of Isoniazid in Aqueous Medium. Water Air Soil Pollut..

[25] Sachan R.S.K., Mistry V., Dholaria M., Rana A., Devgon I., Ali I., Iqbal J., Eldin S.M., Mohammad Said Al-Tawaha A.R., Bawazeer S. (2023). Overcoming Mycobacterium tuberculosis Drug Resistance: Novel Medications and Repositioning Strategies. ACS Omega.

[26] Thangrongthong S., Ladda B., Sittisom P. (2025). A Review of Antibiotic Contamination in Wastewater: Sources, Impacts, and Microbial Bioremediation Techniques. Water Environ. Res..

[27] Hejna M., Kapuścińska D., Aksmann A. (2022). Pharmaceuticals in the Aquatic Environment: A Review on Eco-Toxicology and the Remediation Potential of Algae. Int. J. Environ. Res. Public Health.

[28] Lahlou A., Benlamkaddem S., Berdai M.A., Harandou M. (2019). Seizures following Intoxication with a Common Antituberculosis Drug. Case Rep. Pediatr..

[29] Komai T., Sumitomo S., Teruya S., Fujio K. (2018). Rhabdomyolysis Induced by Isoniazid in a Patient with Rheumatoid Arthritis and End-stage Renal Disease: A Case Report and Review of the Literature. Intern. Med..

[30] Li X., Shen X., Jiang W., Xi Y., Li S. (2024). Comprehensive review of emerging contaminants: Detection technologies, environmental impact, and management strategies. Ecotoxicol. Environ. Saf..

[31] Bankole O.E., Verma D.K., Chávez González M.L., Ceferino J.G., Sandoval-Cortés J., Aguilar C.N. (2022). Recent trends and technical advancements in biosensors and their emerging applications in food and bioscience. Food Biosci..

[32] Rozman M., Štukovnik Z., Sušnik A., Pakseresht A., Hočevar M., Drobne D., Bren U. (2022). A HepG2 Cell-Based Biosensor That Uses Stainless Steel Electrodes for Hepatotoxin Detection. Biosensors.

[33] Štukovnik Z., Godec R.F., Bren U. (2022). The Use of Yeast Saccharomyces Cerevisiae as a Biorecognition element in the Development of a Model Impedimetric Biosensor for Caffeine Detection. Acta Chim. Slov..

[34] Vanderwaeren L., Dok R., Voordeckers K., Nuyts S., Verstrepen K.J. (2022). Saccharomyces cerevisiae as a Model System for Eukaryotic Cell Biology, from Cell Cycle Control to DNA Damage Response. Int. J. Mol. Sci..

[35] Madikizela L., Chimuka L. (2017). Simultaneous determination of naproxen, ibuprofen and diclofenac in wastewater using solid-phase extraction with high performance liquid chromatography. Water SA.

[36] Li K., Zhang M., Ye X., Zhang Y., Li G., Fu R., Chen X. (2021). Highly sensitive and selective detection of naproxen via molecularly imprinted carbon dots as a fluorescent sensor. RSC Adv..

[37] Hernández-Tenorio R., Guzmán-Mar J.L., Hinojosa-Reyes L., Ramos-Delgado N., Hernández-Ramírez A. (2021). Determination of pharmaceuticals discharged in wastewater from a public hospital using LC-MS/MS technique. J. Mex. Chem. Soc..

[38] Azizi N., Hallaj T., Samadi N. (2022). A turn off–on fluorometric and paper-based colorimetric dual-mode sensor for isoniazid detection. Luminescence.

[39] Bhalla N., Jolly P., Formisano N., Estrela P. (2016). Introduction to biosensors. Essays Biochem..

[40] Štukovnik Z., Bren U., Rozman M. (2021). Model Electrochemical Biosensor for the Detection of Methanol in Aqueous Solutions with Yeast Cells. Acta Chim. Slov..

[41] Štukovnik Z., Fuchs-Godec R., Bren U. (2023). Nanomaterials and Their Recent Applications in Impedimetric Biosensing. Biosensors.

[42] Štukovnik Z., Bren U. (2022). Recent developments in electrochemical-impedimetric biosensors for virus detection. Int. J. Mol. Sci..

[43] Fukuda N. (2023). Apparent diameter and cell density of yeast strains with different ploidy. Sci. Rep..

[44] Becerril Ortiz M.E., Ramírez García J.J. (2025). Review of physicochemical processes of naproxen and its byproduct toxicity in aquatic environment. Environ. Technol. Rev..

