# Supplementary material for: Rapid Eukaryotic Impedimetric Biosensing of Naproxen and Isoniazid: A Proof-of-Concept for Acute Toxicity Monitoring
Source: Biosensors (Basel). 2026 May 20;16(5):298. doi: 10.3390/bios16050298 (PMC13204383; doi:10.3390/bios16050298)
Supplement: Supplementary file 1 [file biosensors-16-00298-s001.zip › biosensors-4276925-SI.pdf]

Supplementary Materials

Rapid Eukaryotic Impedimetric Biosensing of Naproxen and Isoniazid: A Proof-of-Concept for Acute Toxicity Monitoring

Zala Štukovnik [1], Nik Perko [1] and Urban Bren [1,2,3,*]

[1] Faculty of Chemistry and Chemical Engineering, University of Maribor, Smetanova ulica 17, 2000 Maribor, Slovenia; zala.stukovnik1@um.si (Z.Š.); nik.perko1@student.um.si (N.P.)
[2] Faculty of Mathematics, Natural Sciences and Information Technologies, University of Primorska, Glagoljaška ulica 8, 6000 Koper, Slovenia
[3] Institute of Environmental Protection and Sensors, Beloruska ulica 7, 2000 Maribor, Slovenia
* Correspondence: urban.bren@um.si

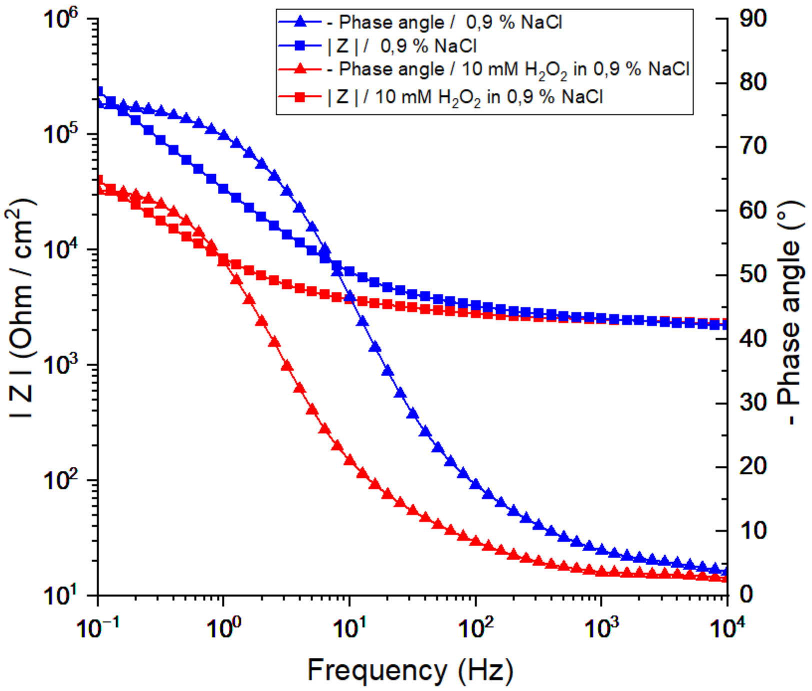

Figure S1. shows the Bode diagram with impedance and phase spectra, depicting the system's response to the addition of 0.9% NaCl (blue curve) and 10 mM H_2O_2 (red curve).

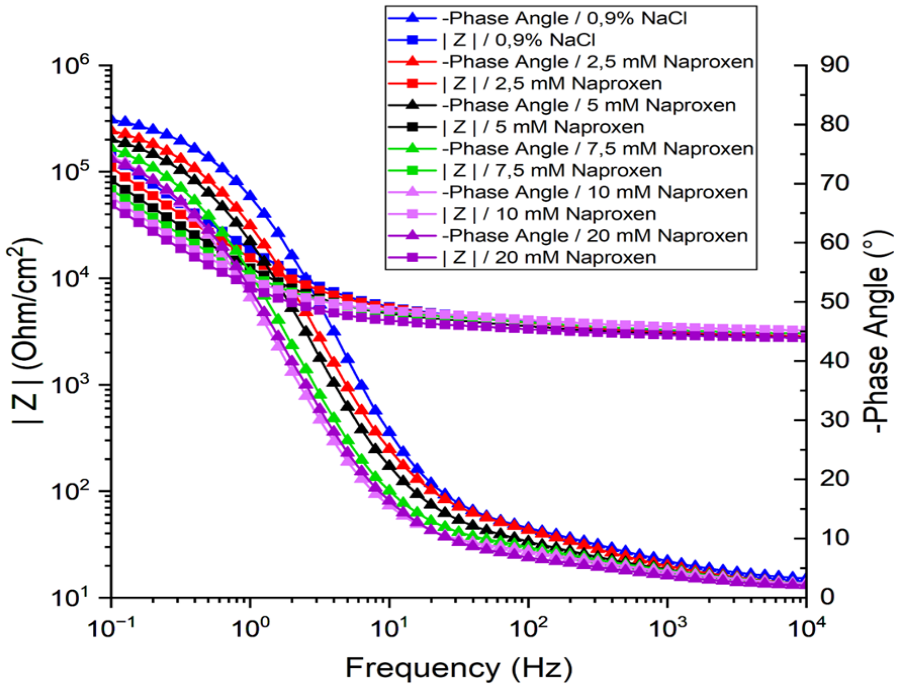

Figure S2. shows the Bode diagram with impedance and phase spectra, depicting the system's response to the addition of 0.9% NaCl (blue curve) and naproxen solutions of varying concentrations (colored curves).

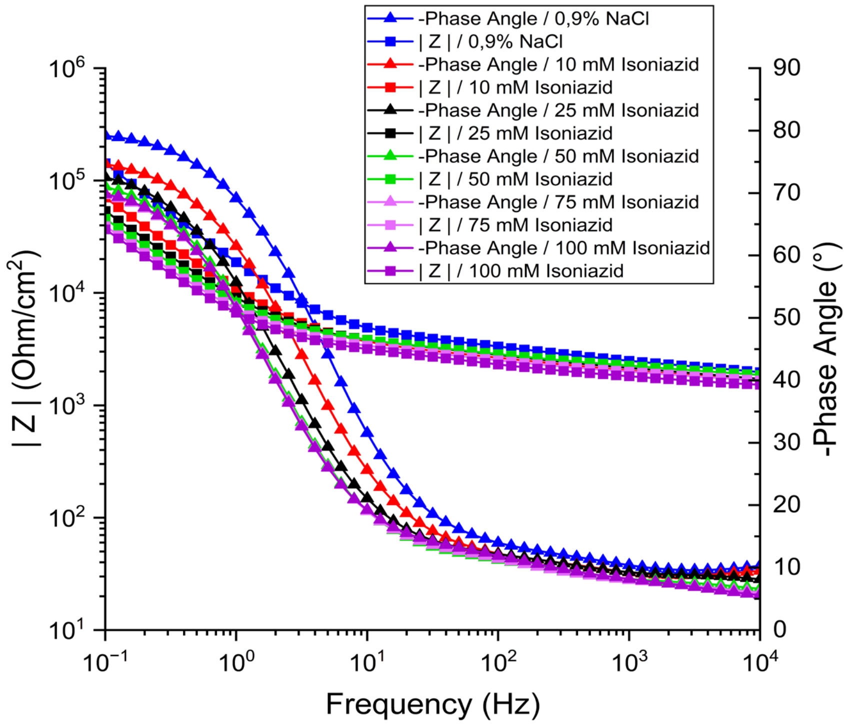

Figure S3. depicts the Bode diagram with impedance and phase spectra, showing the system's response to the addition of 0.9% NaCl (blue curve) and naproxen solutions of varying concentrations (represented by differently colored curves).
